# In Silico Approaches Targeting Quorum-Sensing Inhibition in *Pseudomonas aeruginosa*: A Systematic Review

**DOI:** 10.3390/molecules31162887

**Published:** 2026-08-19

**Authors:** Yeimy Rojas, Cristian Sillagana-Verdezoto, Jacobus de Waard, Cristina Quiroga

**Affiliations:** 1 Carrera de Biotecnología, Facultad de Ciencias de la Vida, Universidad Regional Amazónica Ikiam, Tena 150150, Ecuador; yeimy.rojas@ikiam.edu.ec; 2 Instituto Venezolano de Investigaciones Científicas, Caracas 1020A, Venezuela; 3 Master’s Program in Innovation in Biosciences and Bioengineering, Faculty of Engineering, Universidad San Sebastián, Concepción 4081339, Chile; csillaganav@correo.uss.cl; 4 Facultad de Medicina, Universidad Espíritu Santo, Samborondón 092301, Ecuador; jacobusdeward@gmail.com; 5 Biogeography and Spatial Ecology Research Group, Life Sciences Faculty, Universidad Regional Amazónica Ikiam, Tena 150150, Ecuador

**Keywords:** biofilm, molecular docking simulation, virulence factors, phytochemicals, synthetic drugs

## Abstract

*Pseudomonas aeruginosa* (PA) is a clinically relevant opportunistic pathogen whose persistence and antimicrobial tolerance are largely driven by biofilm formation and quorum-sensing (QS)-regulated virulence. Targeting QS has therefore emerged as an antivirulence strategy that attenuates pathogenicity without exerting strong selective pressure on bacterial growth. This systematic review (2020–2024) analyzes recent advances in the identification of QS inhibitors against PA, emphasizing studies that integrate in silico approaches. Forty-six studies met the inclusion criteria. All employed molecular docking, and 36.9% (*n* = 17) incorporated molecular dynamics simulations. While valuable for initial detection, these computational predictions have inherent limitations in accurately estimating binding energy and conformational dynamics, requiring empirical validation to confirm actual biological activity. Approximately one-fifth of the studies were exclusively computational, whereas the remainder combined in silico screening with in vitro and, in some cases, in vivo assays. The most frequently investigated QS regulators were LasR, PqsR, and RhlR, alongside additional virulence-associated proteins. The evaluated compounds encompassed phytochemicals, synthetic molecules, nanomaterials and natural product-derived compounds, several of which demonstrated experimental evidence of biofilm attenuation and reduction in QS-regulated virulence factors. Overall, the findings highlight the value of integrating computational and experimental strategies to rationally prioritize antivirulence candidates. However, the intrinsic complexity and redundancy of the QS network suggest that future research should increasingly focus on multitarget approaches and on the exploration of chemically diverse and previously underexplored compound libraries to improve efficacy against PA biofilms.

## 1. Introduction

*Pseudomonas aeruginosa* (PA) is a ubiquitous opportunistic pathogen of major clinical relevance, frequently associated with chronic respiratory infections, nosocomial infections, and persistent infections in immunocompromised patients, including individuals with cystic fibrosis, severe burns, organ transplants, or undergoing oncological treatments [1]. Beyond clinical environments, water systems have been identified as important environmental reservoirs and transmission sources of PA, harboring strains characterized by enhanced motility, increased elastase activity, antimicrobial resistance genes, and a high capacity for biofilm formation [2,3]. In response to the growing burden of antimicrobial resistance, particularly to carbapenems, the World Health Organization has classified PA as a critical priority pathogen [4,5].

The remarkable persistence of PA in both clinical and environmental contexts is closely linked to its ability to form biofilms, which are complex multicellular communities embedded in a highly hydrated extracellular polymeric matrix [6,7]. This matrix, mainly composed of exopolysaccharides, proteins, and extracellular DNA, promotes surface adhesion and metabolic cooperation while conferring substantial protection against antimicrobial agents and host immune defenses [1,8]. In PA, the biofilm matrix is primarily constituted by the exopolysaccharides Psl, Pel, and alginate, which play distinct roles in initial attachment, structural maturation, and biofilm stability [9,10,11]. In particular, alginate confers viscoelastic properties that exacerbate disease severity, especially in cystic fibrosis patients, thereby contributing to antimicrobial tolerance and chronic infection persistence [7,12].

Biofilm development and maturation are tightly regulated by quorum-sensing (QS) systems, which enable bacterial populations to coordinate gene expression in response to cell density [13,14]. In PA, QS regulation is mediated by a highly interconnected network composed mainly of the Las and Rhl systems, based on acyl-homoserine lactone (AHL) autoinducers, and the PQS/HHQ system controlled by the transcriptional regulator PqsR [15,16,17]. Together, these systems orchestrate the expression of numerous virulence determinants, including elastases, pigments, biosurfactants, and key components of the biofilm matrix, highlighting QS as a central regulatory hub in PA pathogenicity [18].

Given the central role of QS in virulence regulation, QS inhibition has emerged as a promising antivirulence strategy aimed at attenuating pathogenic fitness without exerting strong selective pressure on bacterial growth [6,19]. Proposed approaches include inhibition of autoinducer synthesis, enzymatic degradation of signaling molecules, and competitive or allosteric blockade of QS regulators such as LasR, RhlR, and PqsR [20]. However, the development of effective QS inhibitors remains challenging due to the hierarchical and redundant organization of the QS network, as well as limitations related to compound stability, specificity, and experimental validation [7,9].

In this context, in silico strategies have progressively evolved from auxiliary tools into a central component of antivirulence drug discovery pipelines [21,22]. Computational methodologies, such as virtual screening (VS), molecular docking, structural modeling, and molecular dynamics simulations (MDs), provide a rational framework to explore large and chemically diverse compound libraries, identify favorable protein–ligand interaction patterns, and prioritize candidates based on predicted binding affinity and structural complementarity [21,23,24]. Importantly, these approaches enable the parallel evaluation of multiple QS regulators, thereby supporting the identification of compounds with potential multitarget activity within the highly interconnected QS network of PA [25,26].

From a translational perspective, in silico screening represents an initial prioritization step rather than a definitive validation of biological activity. Compounds identified through docking and MD simulation constitute a reduced and rationally selected subset that must subsequently undergo experimental validation, including in vitro assays of biofilm formation, QS-regulated virulence phenotypes, and gene expression analyses [27]. Ultimately, the most promising candidates should be evaluated under conditions that better mimic mature biofilms and complex infection environments, as well as explored within multitarget or combination-based strategies to overcome compensatory QS pathways.

Despite the growing number of studies applying computational approaches to target QS in PA, a comprehensive and systematic synthesis integrating in silico methodologies with experimental validation remains limited. Consequently, a critical analysis of methodological trends, molecular targets, and compound classes is required to guide future antivirulence discovery efforts.

Therefore, the aim of this systematic review is to provide a qualitative synthesis of recent studies (2020–2024) that employ in silico methodologies to identify chemical compounds capable of inhibiting QS and QS-regulated biofilm formation in PA. Specifically, this qualitative analysis emphasizes computational strategies, molecular targets, experimental validation pathways, and future multitarget antivirulence approaches.

It is crucial to interpret computational predictions, particularly those derived from molecular docking, with scientific caution. These algorithms are based on thermodynamic approximations of binding free energies and frequently employ rigid or semi-flexible receptor models, failing to fully account for solvent effects, environmental dynamics, and protein conformational plasticity [21,22,26]. Consequently, virtual screening campaigns remain susceptible to generating false positives, in which compounds exhibit high theoretical affinity but lack actual in vitro or in vivo efficacy. Therefore, throughout this review, in silico findings are strictly evaluated as initial predictive hypotheses rather than definitive confirmations of biological activity.

### Quorum-Sensing System in Pseudomonas aeruginosa

**Figure 1:**
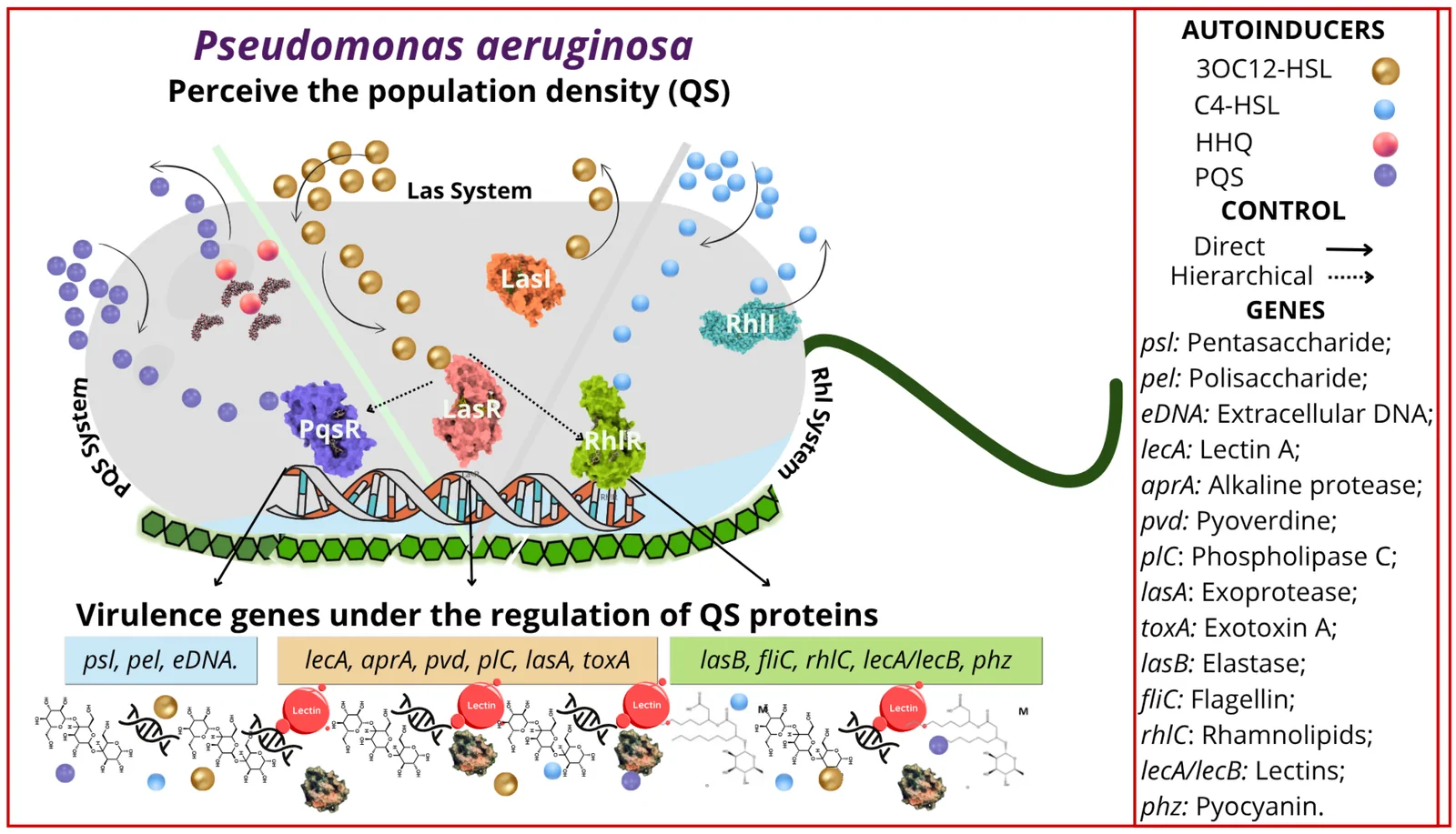
Overview of the quorum-sensing regulatory network controlling virulence gene expression in *Pseudomonas aeruginosa*.

Biofilm development and maturation in PA are regulated by a highly organized QS network that coordinates gene expression in response to cell density and environmental cues [28]. Rather than operating as a single signaling pathway, QS in PA consists of a multilayered and hierarchical regulatory network composed of four interconnected systems: Las, Rhl, PQS, and IQS, which interact through mechanisms of activation and cross-regulation (Figure 1) [16,17,28]. This interconnected architecture allows the bacterium to finely tune virulence factor expression and biofilm development under diverse environmental conditions.

Within this network, the Las system occupies a dominant regulatory position and exerts hierarchical control over the Rhl and PQS systems [29,30], while Rhl functions as a key effector module regulating genes associated with biofilm architecture and dispersal [31]. The PQS system further integrates virulence regulation with iron metabolism and contributes to biofilm maturation [16,32]. In addition, PA employs the Integrated QS (IQS) system, which links environmental stress signals, such as phosphate limitation, to the classical QS circuitry [28,33]. IQS-mediated regulation enables sustained activation of QS-controlled pathways even in the presence of mutations in central regulators such as LasR, a phenomenon frequently observed in clinical isolates. This regulatory plasticity underscores the robustness and redundancy of the QS network and highlights the limitations of single-target inhibition strategies [28,34]. Despite the growing number of studies reporting quorum-sensing inhibitors, in silico strategies actually identify translationally relevant antivirulence candidates or, conversely, generate computational false positives that inadvertently lead to costly and erroneous experimental validation efforts and translation bottlenecks. Therefore, this review not only summarizes recent findings but also evaluates the methodological robustness and predictive value of computational approaches used in QS inhibitor discovery.

## 2. Results

### 2.1. General Overview of Included Studies

To ensure the full reproducibility of this systematic review, a comprehensive literature search was conducted in PubMed, Scopus, and Web of Science. The final search across all databases was performed on 30 October 2024. The search strategy used combinations of MeSH terms and free-text keywords linked by Boolean operators. The exact search string applied was: (“*Pseudomonas aeruginosa*” OR “*P. aeruginosa*”) AND (“quorum sensing” OR “quorum quenching” OR “biofilm*” OR “antibiofilm” OR “virulence”) AND (“in silico” OR “docking” OR “virtual screening” OR “computational”). No language restrictions were applied during the initial search, but time filters were set to retrieve articles published between 1 January 2020 and the exact date of the search.

The systematic literature search identified a total of 527 publications between 2020 and 2024 that were potentially related to the defined search terms and keywords. The selection and review of articles were performed independently by two authors. Any discrepancies regarding the application of the exclusion criteria were resolved through discussion until a mutual consensus was reached. After applying the predefined inclusion and exclusion criteria, 45 studies were retained for qualitative synthesis in this review (Table 1). The temporal distribution of the included studies revealed a marked increase in publications over time, with 39.1% (*n* = 18) published in 2024, followed by 21.7% (*n* = 10) in 2023, 17.4% (*n* = 8) in 2022, 15.2% (*n* = 7) in 2021, and 6.6% (*n* = 3) in 2020.

Table 1 summarizes the main characteristics of the included studies. Of the total number of studies analyzed, a small proportion (19.6%, *n* = 9) were based exclusively on in silico methodologies. while the remaining studies combined computational approaches with experimental microbiological, molecular, and/or chemical assays. Among studies that included in vitro validation, the reference strain PAO1 was the most frequently employed (58.4%, *n* = 27), followed by ATCC strains (10.8%, *n* = 5) and clinical isolates (5.6%, *n* = 3).

**Figure 2:**
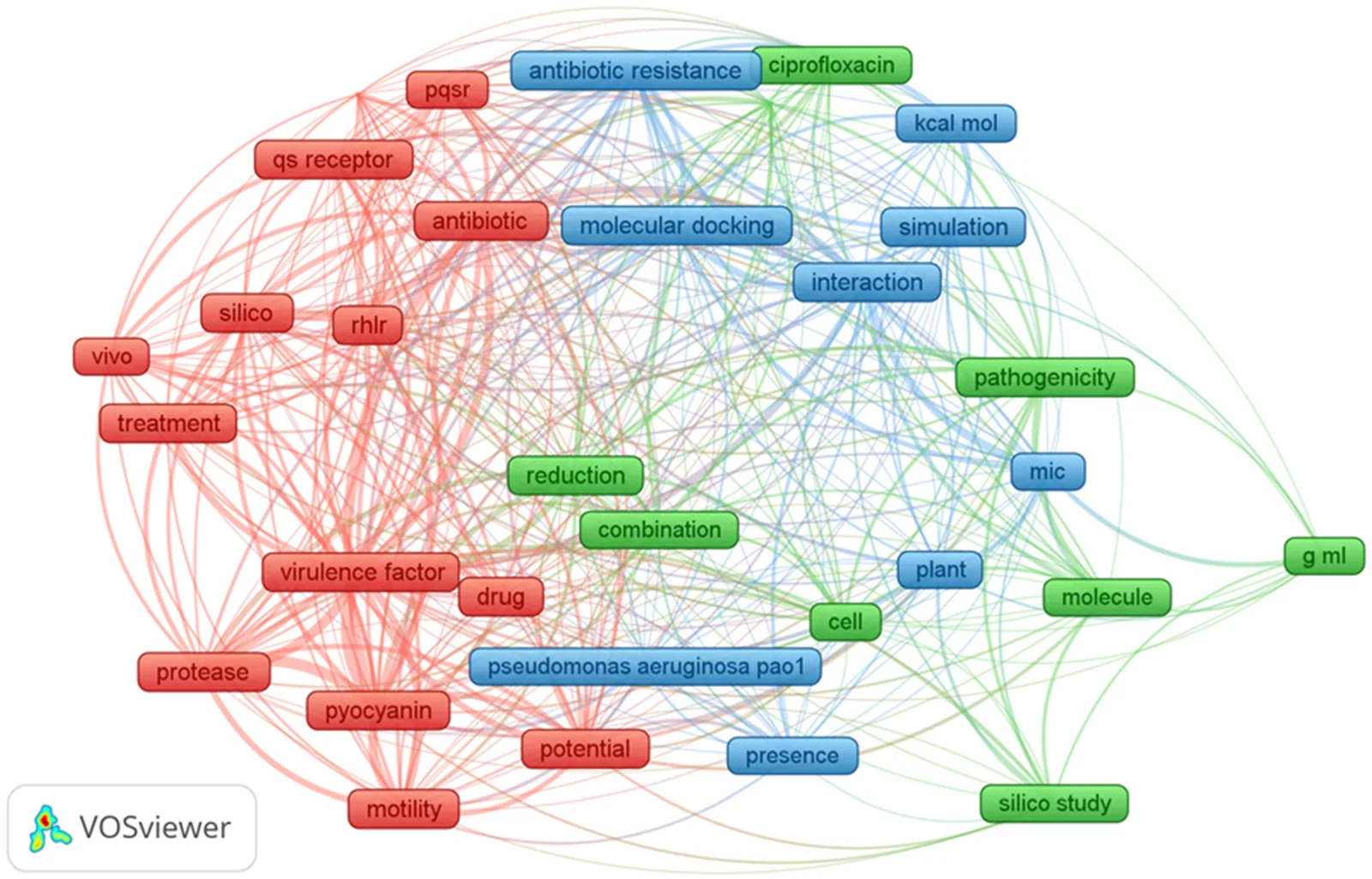
Keyword co-occurrence network in quorum-sensing research on *Pseudomonas aeruginosa*. The groups represent terms associated with QS and virulence (red), computational methodologies (blue), and experimental evaluation and compound origin (green).

Keyword co-occurrence analysis (Figure 2), performed using keywords with a minimum occurrence frequency of five, revealed three major thematic clusters. The first cluster comprised terms associated with QS and virulence, including QS receptors, motility, and metabolite production. The second cluster grouped keywords related to computational methodologies, such as molecular docking, molecular simulations, protein–ligand interactions, and energetic parameters. The third cluster included terms linked to experimental evaluation and compound origin, including cellular assays, minimum inhibitory concentration, and plant-derived molecules. Together, these clusters reflect the integrative computational–experimental nature of the analyzed studies.

### 2.2. Computational Approaches and Validation Strategies

All included studies applied at least one in silico methodology, with docking employed in 100% (*n* = 100) of the analyzed publications. In addition, 36.9% (*n* = 17) of the studies complemented docking analyses with MD to evaluate the stability and persistence of protein–ligand interactions over time. A subset of studies implemented comprehensive computational pipelines combining VS, docking, and MD, and were conducted exclusively as in silico research.

Despite the widespread use of molecular docking across the analyzed studies, significant variability was observed in the implementation of docking protocols. Differences in scoring functions, grid box definitions, ligand preparation procedures, and validation strategies (such as redocking or use of reference ligands) can substantially influence predicted binding affinities and interaction patterns. This methodological heterogeneity limits the direct comparability of results across studies and may contribute to inconsistencies in reported binding energies and ligand rankings.

Among studies that incorporated experimental validation, two main strategies were identified: (a) the use of in silico methodologies as a pre-experimental prioritization tool to select promising candidate compounds prior to laboratory testing, and (b) the application of computational tools as a post-experimental approach to rationalize and interpret observed biological effects.

Regarding docking validation, 67.4% (*n* = 31) of the studies performed redocking using co-crystallized ligands of the selected receptors to assess the reliability of the docking protocol, while an additional 2.6% (*n* = 1) employed experimentally validated receptor inhibitors as reference ligands. The absence of redocking was not considered an exclusion criterion because the purpose of this review was to provide a broad overview of current in silico strategies used in this field rather than to restrict the analysis to studies with a single validation framework. However, the lack of validation procedures may significantly affect the reliability of docking predictions and, therefore, should be considered when interpreting and comparing the reported results.

**Figure 3:**
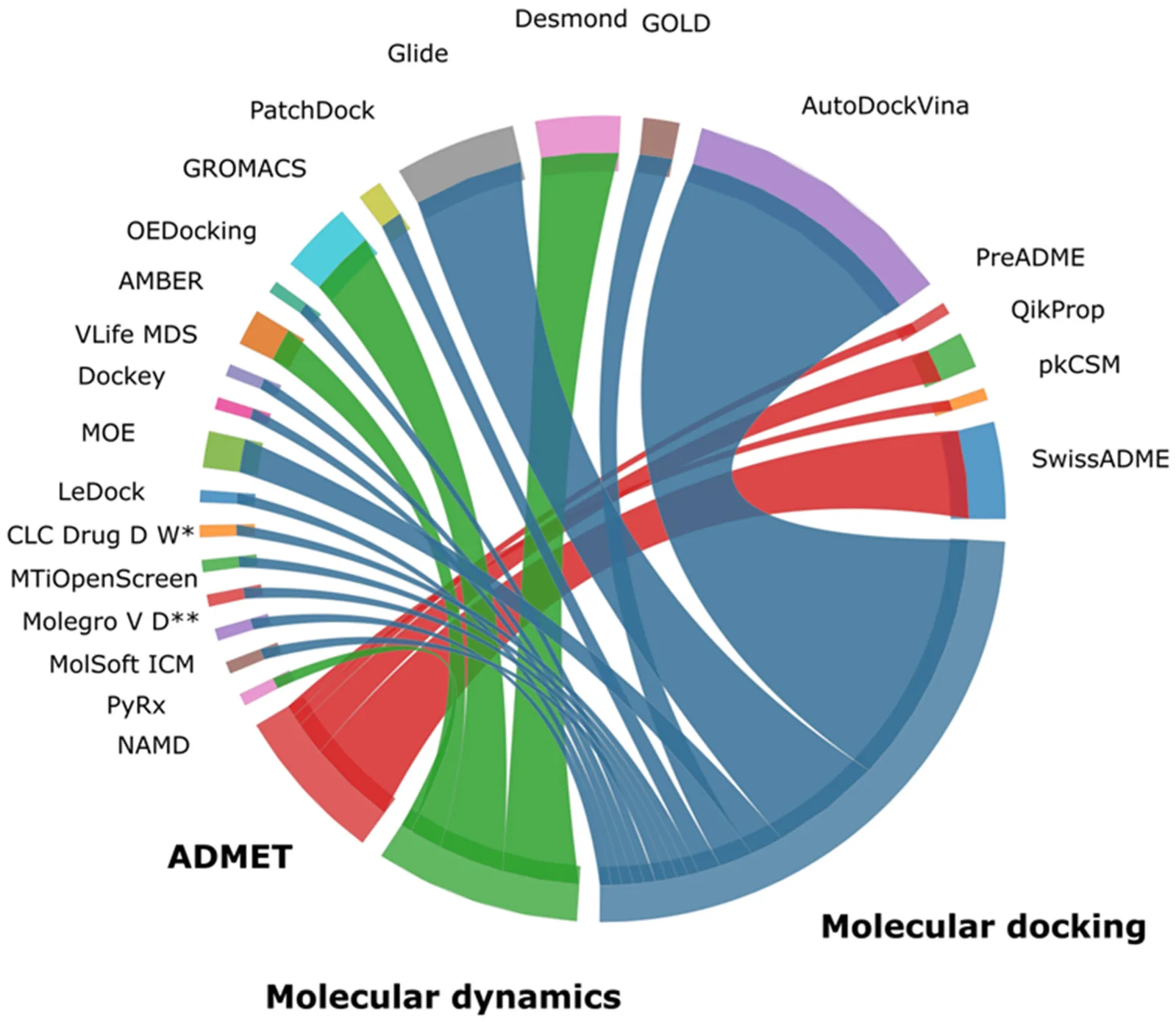
Distribution of bioinformatics tools across molecular docking, molecular dynamics, and ADMET analyses in quorum-sensing studies on *Pseudomonas aeruginosa*. * CLC Drug Discovery Workbench, ** Molegro Virtual Docker.

The computational tools employed across the analyzed studies are summarized in Figure 3. AutoDock Vina 1.2.0 was the most frequently used software for docking (50% of the studies, *n* = 23), Desmond was the most commonly applied platform for MD (43.8%, *n* = 7), and SwissADME was used in 61.5% (*n* = 8) of the studies that evaluated pharmacokinetic and drug-likeness properties.

### 2.3. Molecular Targets and Structural Modeling Strategies

The molecular targets evaluated in the analyzed studies are summarized in Table 2. The most frequently investigated QS receptors were LasR (31.4%, *n* = 32), followed by RhlR (16.7%, *n* = 17), LasI and PqsR (13.7% each, *n* = 14), and QscR and RhlI (3.9%, *n* = 4). In addition to canonical QS receptors, several studies included QS-dependent virulence-related proteins functionally linked to QS but external to the core QS circuitry. These included LasA, LasB, LpxC, PqsA, PqsE, ExoS, ExoT, ExoU, PilY1, and PilT.

It is important to distinguish between canonical quorum-sensing regulators and QS-associated virulence-related proteins. Canonical QS targets include key transcriptional regulators, such as LasR, RhlR, and PqsR, which directly control quorum-sensing signaling pathways. In contrast, other proteins frequently evaluated in the analyzed studies, such as LasB, ExoS, PqsA, and related virulence factors, are indirectly regulated by QS systems and therefore represent secondary targets rather than core components of the QS regulatory network.

For the most commonly studied receptors (LasR, PqsR, and RhlR), multiple crystallographic structures were employed, reflecting structural diversity and conformational variability. Up to ten different crystallographic structures were reported for LasR, and five structures each for PqsR and RhlR. When experimentally determined structures were not available, protein structure prediction strategies were applied, including homology modeling using SWISS-MODEL (for RhlI, RhlR, and PqsA) and artificial intelligence-based structure prediction using AlphaFold (for RhlI and RhlR).

### 2.4. Origin and Chemical Nature of Evaluated Ligands

The evaluated ligands exhibited substantial diversity in terms of origin and method of acquisition (Table 1). A total of 23.9% (*n* = 11) of the studies employed compounds previously reported in the scientific literature. In 26.1% (*n* = 12) of cases, ligands were obtained through chemical synthesis, including alkyl imidazoles, imidazole derivatives, flavonoids, activated furan compounds, cinnamic acid derivatives, catechol–zingerone conjugates, synthetic xanthine derivatives, phenyl oxadiazole sulfoxides, prazosin, zinc oxide nanoparticles (ZnO-NPs), and selenium oxide nanoparticles.

Additionally, 17.4% (*n* = 8) of the studies identified ligands through chromatographic and spectroscopic characterization of plant extracts or biosurfactants produced by *Lactiplantibacillus plantarum.* Furthermore, 21.7% (*n* = 10) of the studies relied on chemical compound databases of medicinal plants, food-derived compounds, or marine metabolites. Finally, 10.9% (*n* = 5) of the studies evaluated commercially available plant-derived natural compounds, including psoralen, vitamins E and K_1_, emodin, gallic acid, 6-gingerol, and coumarin.

Table 3 summarizes the 20 most promising ligands reported as potential QS inhibitors based on docking binding affinity values. Among these, 3,3′-dihydroxy-4,5-dimethoxybibenzyl, lupiwighteone, shinpterocarpin, and (R)-glabridin exhibited the most negative binding energies −1 (−14.5, −13.3, −13.3, and −13.2 kcal/mol, respectively). These compounds consistently showed π–π interactions with key LasR residues, including Phe101, Tyr64, and Trp88. Overall, LasR was the most frequently targeted receptor (75%, *n* = 15), followed by QscR and PqsA (10% each, *n* = 2).

However, it is important to note that docking-derived binding affinity values represent theoretical predictions of protein–ligand interactions and do not necessarily correlate with in vitro or in vivo biological activity. Therefore, these results should be interpreted as an initial prioritization step rather than definitive evidence of QS inhibition.

### 2.5. Experimental Validation of Computational Predictions

The experimental methodologies employed across the analyzed studies are summarized in Table 4 and included determination of minimum inhibitory concentration (MIC) and minimum bactericidal concentration (MBC), biofilm inhibition assays using crystal violet and Congo red staining, gene expression analysis by RT-qPCR, and microscopy-based techniques for biofilm structural evaluation. These microscopy approaches included optical microscopy, confocal laser scanning microscopy, and scanning electron microscopy, which enabled visualization of biofilm architecture and assessment of inhibitor efficacy.

Additional assays evaluated the production of exopolysaccharides and QS-regulated virulence factors, including pyocyanin, pyoverdine, and proteases. In studies focused on compound identification and characterization, chromatographic and spectroscopic techniques such as gas chromatography–mass spectrometry (GC–MS), high-performance liquid chromatography (HPLC), thin-layer chromatography (TLC), and nuclear magnetic resonance (NMR) spectroscopy were employed.

Table 5 summarizes ligands that combined favorable binding affinity values with experimental evidence. Luteolin and chrysin exhibited the lowest MIC values (0.08 and 0.009 mg/mL, respectively). Rhein showed consistent inhibitory effects on swarming motility and multiple virulence factors, including proteases, LasB elastase, pyocyanin, and rhamnolipids at 0.15 mg/mL. Zinc oxide nanoparticles displayed inhibition levels comparable to rhein when evaluated at 300 mg/mL, while rutin achieved the highest reported biofilm inhibition, reaching 73.7% at 0.25 mg/mL.

## 3. Discussion

### 3.1. Methodological Strategies for the Identification of QS Inhibitors

All the analyzed studies were found to include molecular docking as a computational methodology for predicting protein–ligand interactions associated with QS. The docking process comprises two main stages: predicting the ligand conformation and evaluating its binding affinity [79,80]. In total, 64.1% (*n* = 30) of the studies applied redocking using co-crystallized ligands as a docking validation procedure. However, relying solely on this strategy constitutes a significant methodological limitation. Re-coupling only confirms the algorithm’s ability to reproduce a native conformation within a pre-adapted binding pocket, which introduces a structural bias that does not guarantee predictive accuracy for new and diverse chemotypes. Consequently, achieving genuine predictive reliability requires implementing more rigorous validation frameworks, such as cross-coupling, benchmarking with ligands of known activity, or dynamic analysis of protein–ligand complexes [81].

Molecular docking was applied as both a pre-experimental and post-experimental methodology. The pre-experimental approach, known as structure-based virtual screening, has the main advantage of being able to identify new chemotypes from extensive chemical libraries for a target of interest, which are generally not related to ligands previously characterized for that target [82]. However, the success and interpretability of both approaches depend heavily on the crystallographic resolution of the selected receptor. Lower-resolution structures introduce critical uncertainties in the coordinates of side chains and hydration networks, which can compromise the accuracy of the docking by generating distorted binding cavities, unreliable ligand conformations, and misleading affinity scores [83,84,85].

In the reviewed studies, most docking analyses were based on experimentally determined protein structures, whose crystallographic resolution is a criterion to consider in the interpretation of the results (Table 2). Resolution differences directly determine the accuracy of the coupling: high-resolution structures provide precise atomic coordinates and define essential hydration networks, while lower resolutions introduce ambiguities in side-chain orientations and pocket geometries, which can lead to unreliable ligand conformations and inaccurate scoring. The literature indicates that resolutions below 1.2 Å represent an ideal scenario for docking small molecules, while most available crystallographic structures have resolutions between 1.5 and 2.5 Å, with values below 2.2 Å generally accepted for practical docking applications [86,87,88].

In cases where crystallographic structures were unavailable, studies resorted to homology-based structural modeling, primarily using SWISS-MODEL [89], or to predictions based on artificial intelligence, such as AlphaFold [90]. Homology-based modeling depends on the availability and quality of a template structure and is most reliable when sequence identity exceeds approximately 30–35%, with the main errors being attributable to imprecise alignments and regions of low structural conservation [91]. In contrast, AI-based approaches do not require direct templates and use large-scale evolutionary information to generate structural models [92]. However, the literature emphasizes that both approaches require subsequent structural evaluation using metrics and validation tools before their application in docking studies [93,94].

### 3.2. Inhibitors and Molecular Targets Associated with QS

The most studied proteins were LasR, PqsR, LasI, and RhlR, which are directly involved in the PA chemosystem. LasI participates in the functional activation of the LasR transcriptional receptor [95], triggering biofilm formation and the expression of various virulence factors, such as elastase, proteases, pyocyanin, lectins, rhamnolipids, and toxins [96]. RhlR, for its part, is a transcriptional regulator of the Rhl system that controls the expression of genes associated with virulence factors, exoproduct production, and biofilm formation, and can activate gene expression even in the absence of its autoinducer C4-HSL [97]. Furthermore, PqsR is a quinolone-signaled transcriptional regulator that controls transcription of the pqsABCDE operon, which is responsible for the production of quinolone molecules involved in pathogenicity, iron chelation, and cytotoxicity in PA [98]. To successfully translate inhibitors against these essential targets into clinical practice, current research must integrate structural predictions with established pharmacological frameworks. Beyond binding efficacy, it is crucial to evaluate candidates for their optimal physicochemical properties, drug similarity, and robust ADMET (absorption, distribution, metabolism, excretion, and toxicity) profiles in order to eliminate compounds that, despite high theoretical affinities, lack true therapeutic viability.

To analyze the biological relevance of the inhibitors identified through in silico studies, priority was given to discussing those compounds that, in addition to exhibiting high predicted binding affinities, had experimental evidence of biological activity. In this regard, it has been described that QS inhibitors must meet certain general criteria, including small molecular size, specificity for particular QS regulators, chemical stability, and resistance to degradation by host metabolic systems, as well as, ideally, a length greater than that of native AHL [99]. Furthermore, previous studies have documented that QS inhibitors can act through various mechanisms, including inhibition of signal molecule synthesis, enzymatic degradation, competition for receptor sites, blocking the transcriptional activation of QS-regulated genes, and neutralization of inducers by macromolecules such as cyclodextrins [99,100].

Chrysin is a flavonoid found in plants, edible mushrooms, honey, and propolis [101,102]. Luteolin, a potent antioxidant belonging to the flavonoid family, is found in vegetables and edible plants in the form of glycosides [103]. Rutin, also a flavonol glycoside, is associated with a range of pharmacological activities, including antioxidant, cytoprotective, vasoprotective, anticancer, neuroprotective, and cardioprotective properties [104,105,106,107]. These compounds have been shown to exhibit experimental evidence of biological activity against PA and high in silico binding affinity to chemoreceptors, which is associated with inhibition of bacterial growth, as well as a reduction in swimming and swarming motility and protease activity.

Experimental studies have demonstrated that flavonoids act as modulators of the chemoreceptor system in PA. These compounds exert their effect through a mechanism consistent with the allosteric inhibition of LuxR-type receptors, such as LasR and RhlR, interfering with the binding of the regulatory complex to DNA and reducing the transcriptional activation of virulence-associated genes. Consequently, a decrease in the expression of QS-regulated factors has been observed, including the production of virulence metabolites and biofilm formation, without directly affecting bacterial growth [108].

Furthermore, ZnO-NPs were observed to have the potential to inhibit both LasR and LasI and exhibited high binding affinity in silico (Table 5). Experimental studies have demonstrated that ZnO-NPs act as effective inhibitors of the QS system in PA. At concentrations better than the MIC, ZnO-NPs significantly reduce the production of QS-regulated virulence factors, including rhamnolipids, pyocyanin, pyoverdin, hemolysins, elastase, and proteases, without significantly affecting bacterial growth. At the molecular level, a decrease in the expression of key QS regulatory genes has been observed, including the lasI/lasR, rhlI/rhlR, and pqsA/pqsR systems, suggesting global interference in QS signaling circuits [109].

### 3.3. Limitations and Future Prospects

The present review was primarily based on studies employing in silico approaches, particularly molecular docking, as the central methodology for the identification of potential inhibitors of the QS system in PA. One limitation of this approach is that docking-derived affinity values allow only structural-level comparisons of interactions and do not directly reflect biological activity or experimentally observed inhibitory efficacy. Consequently, in silico results should be interpreted as a compound prioritization stage rather than as conclusive evidence of QS inhibition.

Docking methods are particularly prone to false positives, as high binding affinity scores do not necessarily correlate with in vitro or in vivo activity. This limitation highlights the need for cautious interpretation of ranked ligand lists, such as those presented in Table 3.

Although in silico approaches are widely used in QS inhibitor discovery, their overall success in accurately predicting biologically active compounds remains uncertain. The current literature lacks consistent statistical evidence demonstrating their reliability in identifying true inhibitors across diverse chemical spaces.

Molecular dynamics simulations can provide additional insights into the stability of protein–ligand complexes; however, their predictive reliability depends strongly on methodological choices, including the selection of force fields (such as AMBER, CHARMM, or OPLS) and solvent models, including explicit water models (e.g., TIP3P or TIP4P) and implicit continuum approaches (e.g., Generalized Born or Poisson–Boltzmann methods) [25]. These parameters directly influence interaction dynamics and endpoint binding free-energy calculations, such as MM/PBSA or MM/GBSA protocols; yet, they are frequently reported inconsistently across studies, thereby limiting comparability and reproducibility.

For example, implicit solvent approaches may inadequately represent structural water-mediated interactions and long-range electrostatic effects within protein–ligand binding pockets, potentially influencing the accuracy of docking and molecular dynamics predictions [26]. Similarly, inadequate force field parameterization for novel ligand chemotypes may affect the structural stability and conformational behavior of flexible protein regions during simulations [22,26]. Because these methodological parameters are frequently reported inconsistently or omitted in the reviewed studies, comparability and reproducibility between studies remain limited.

Furthermore, molecular docking relies on static receptor models and does not fully account for protein conformational dynamics or the competitive binding environments characteristic of experimental conditions, including inhibitory concentrations and molecular competition. Most experimental studies also evaluated bacterial inhibition in planktonic states or early-stage biofilms, limiting the direct extrapolation of in silico predictions to mature biofilms protected by extracellular matrix barriers. Given that QS in Pseudomonas aeruginosa operates as an integrated regulatory network, inhibition of a single target does not necessarily guarantee global disruption of virulence-associated pathways. Therefore, while in silico approaches remain valuable for prioritizing candidate compounds, their effectiveness in identifying true QS inhibitors remains highly dependent on methodological rigor and subsequent experimental validation.

Beyond these study-specific limitations, a methodological limitation of this systematic review should also be acknowledged. The exclusive inclusion of open-access articles was due to institutional access restrictions and budgetary limitations associated with the paywalled literature. Although this criterion may introduce potential selection bias, its overall impact on the final synthesis is considered limited. The extensive volume of the open-access literature published between 2020 and 2024 still provides a representative cross-sectional overview of current computational methodologies and experimental validation strategies in the field.

From a future perspective, the analyzed results suggested that strategies exclusively targeting a single QS regulator may be insufficient in light of the regulatory complexity of PA. In this context, approaches prioritizing the identification of compounds capable of interacting with multiple components of the QS system or acting in a complementary manner on different signaling pathways, may offer greater efficacy in biofilm inhibition. Likewise, the exploration of under-studied chemical libraries, including natural compounds not previously evaluated against PA, represents a relevant opportunity to identify new chemotypes with alternative mechanisms of action. The prioritization of molecules without prior bacterial exposure may reduce the likelihood of adaptive responses and broaden the spectrum of candidates with QS inhibitory potential, thereby strengthening future strategies for the control of bacterial virulence.

## 4. Materials and Methods

### 4.1. Literature Search Strategy

This study was designed as a systematic review with qualitative and descriptive synthesis focused on the application of in silico methodologies to identify inhibitors of QS-regulated biofilm formation in PA. A systematic literature search was conducted to identify relevant studies published between January 2020 and December 2024 that applied computational approaches, either exclusively or in combination with experimental validation, to evaluate compounds with potential antibiofilm activity against PA.

The literature search was performed using the following databases: Scopus, Web of Science, PubMed, ScienceDirect, DOAJ, and Redalyc. The final search across all databases was performed on 30 October 2024. The search strategy used combinations of MeSH terms and free-text keywords linked by Boolean operators. The exact search string applied was: (“*Pseudomonas aeruginosa*” OR “*P. aeruginosa*”) AND (“quorum-sensing” OR “quorum quenching” OR “biofilm*” OR “antibiofilm” OR “virulence”) AND (“in silico” OR “docking” OR “virtual screening” OR “computational”). Only peer-reviewed articles published in English or Spanish were considered.

### 4.2. Eligibility Criteria

Studies were included if they met the following criteria: (i) open-access availability; (ii) focus on PA as the target organism; (iii) application of at least one in silico methodology, such as molecular docking, VS, or MD; and (iv) reporting of binding affinity or binding energy values for protein–ligand interactions involving QS regulators or QS-related virulence proteins.

Studies were excluded if their primary objective was vaccine design, RNA-based inhibition strategies, or if they focused on organisms other than PA. Duplicate records identified across databases were removed prior to screening.

The restriction to open-access articles was applied due to accessibility constraints. This criterion may introduce a selection bias by excluding potentially relevant studies; however, it ensured full access to methodological details required for consistent data extraction and comparative analysis.

### 4.3. Study Selection Process

This systematic review followed the PRISMA 2020 guidelines for the identification, selection, eligibility, and inclusion of studies [110]. A formal protocol was not registered in PROSPERO, as we focused on in silico and preclinical drug discovery rather than clinical outcomes. Furthermore, since standard risk-of-bias tools (e.g., Cochrane) are specifically designed for clinical, epidemiological, or in vivo studies, they were considered inapplicable to our dataset. We substituted a detailed methodology for risk-of-bias assessment during data extraction. This assessment focused on critical computational and structural parameters, including the crystallographic resolution of target proteins and dynamic validation of docking protocols.

**Figure 4:**
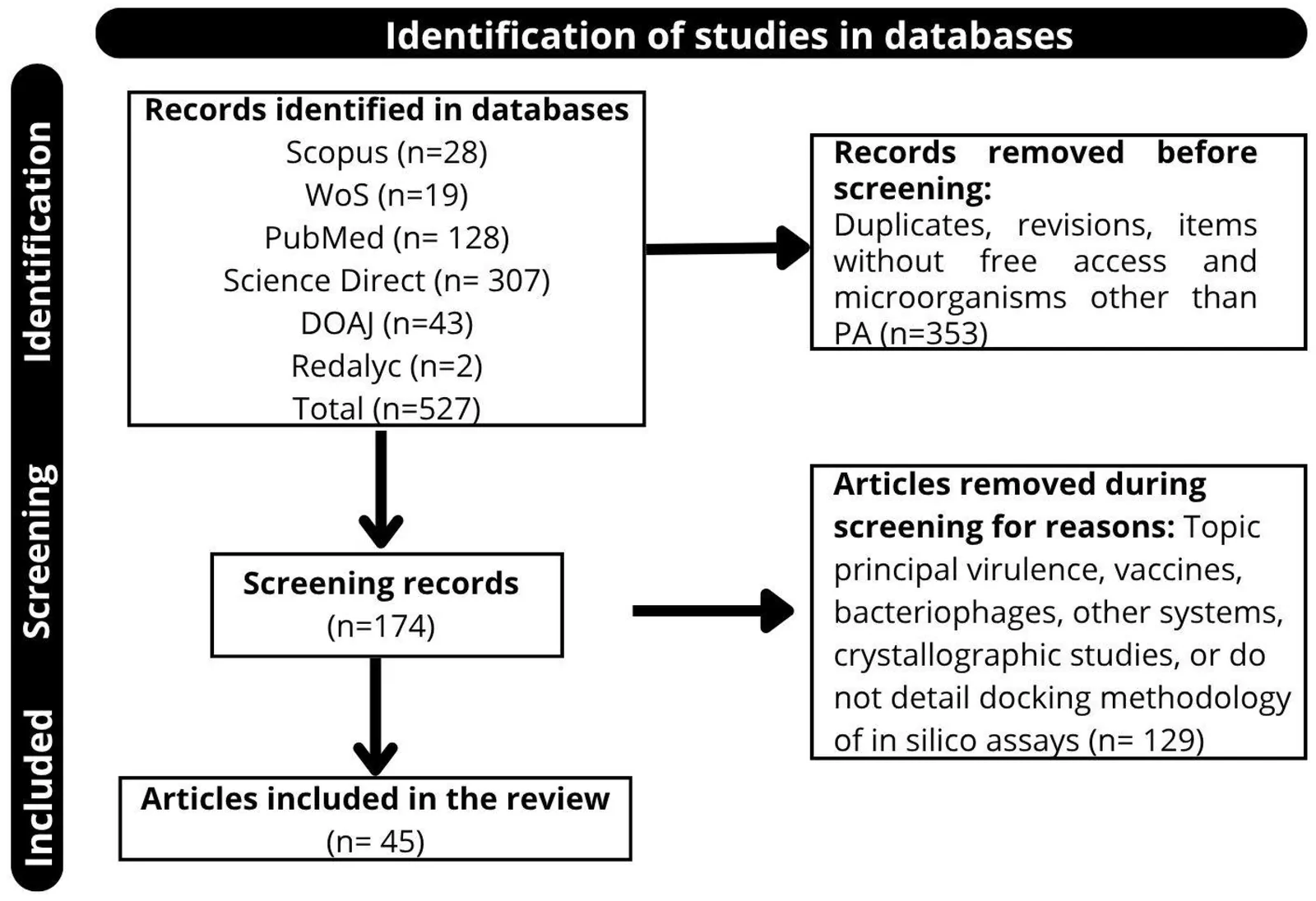
PRISMA flow diagram illustrating the identification, screening, and inclusion of studies on quorum-sensing inhibition in *Pseudomonas aeruginosa*.

All records retrieved from the selected databases were imported and screened by title and abstract to assess relevance. Duplicates were removed prior to screening. Full-text assessment was performed for studies in which the methodological approach was unclear from the abstract. Only studies meeting all inclusion criteria were retained for qualitative synthesis. The complete selection process is illustrated in the PRISMA flow diagram (Figure 4).

Due to the heterogeneity of computational protocols, molecular targets, compound classes, and experimental validation strategies, a quantitative meta-analysis was not performed.

### 4.4. Data Extraction and Qualitative Synthesis

Data extraction was performed manually using a standardized spreadsheet (Microsoft Excel), with one row per included study. The following variables were extracted and categorized into predefined columns: article title, authors, year of publication, compound origin and class, molecular targets, in silico methodologies employed (VS, docking, MD and ADMET), reported binding affinity values, validation strategies (e.g., redocking, reference ligands), computational software and web servers used, experimental methodologies (in vitro or in vivo), bacterial strains employed, and key experimental outcomes.

A descriptive and comparative analysis was conducted to summarize publication trends, distribution of computational approaches, frequency of molecular targets, origin of evaluated compounds, and experimental validation strategies. Percentages and frequencies were calculated to support the classification of studies and to generate summary tables and figures. Additionally, keyword co-occurrence analysis was performed based on reported keywords to identify major thematic clusters related to QS, computational methodologies, and experimental validation.

## 5. Conclusions

Based on the literature reviewed, this study suggests that a major challenge in discovering quorum-sensing inhibitors lies not necessarily in a lack of candidate molecules, but rather in the methodological design of screening strategies. Current approaches often rely on single-target docking predictions that may not fully reflect the complexity of the regulatory network controlling virulence in Pseudomonas aeruginosa. Future progress in antivirulence therapy would greatly benefit from integrating bio-based computational screening, multi-target validation, and infection-relevant experimental models. In addition to identifying further high-affinity ligands, the field should emphasize predictive experimental frameworks capable of distinguishing true anti-biofilm candidates from computational false positives.

## Figures and Tables

**Table 1:** Characteristics of studies targeting quorum-sensing-related proteins in *Pseudomonas aeruginosa*.

Reference	Strain Bacterial	Type of Study	Computational Method	Drug Target	Origin of Ligands
[35]	PAO1	1, 2	Docking	LasR, LasI, RhlR, RhlI	Literature
[36]	PA ATCC 27, 853	1, 2	VS, docking	LasR, PqsR, LasI, AcrB, MexB, OprN, OprJ, RhlI	*Tabernaemontana crassa* extract
[37]	QS protein expression from PA in *E. coli* DH5α	1, 2	Docking	LasR, RhlR	Literature
[38]	PA (Gene Bank Accession Number: OK355439)	1, 2	Docking, MD	LasR, LpxC	*Christella dentata* extract
[39]	PA strains: PA14-WT, PA14-R3, PAO1-WT, PAO1-CTX, ATCC 10145	1, 2	Docking	LasR, PqsA, PsqE, PqsR	Literature
[40]	PAO1	1, 2	Docking	LasR	Synthesis of alkyl imidazoles
[41]	PA	1, 2	Docking	LasI, LasR, RhlI, RhlR	Nanoparticles
[42]	PA	1, 2	Docking	LasR	Synthesis of imidazole derivatives
[43]	PAO1	1, 2	Docking, MD	LasR, Vfr, PqsA, QscR	Literature
[44]	PAO1	1, 2	Docking, MD	LasI, LasR	*Myrtus communis* extract
[45]	PAO1	1, 2	Docking, MD	LasR, PqsR, RhlR	Synthesis of flavonols
[46]	PAO1	1, 2	Docking	LasR, PqsR, RhlR	Compounds synthesized from cinnamic acid
[47]	PAO1	1, 2	Docking, MD	PqsR	*Ferula assafoetida* extract
[48]	PA ATCC 27853	1, 2	Docking, MD	LasR, LasB, LasI, ToxA, AprA	Sunflower seeds oil
[49]	PAO1, PA14, PAO1 ΔlasI, PAO1-ΔrhlI, PAO1-ΔpqsA, 10 clinical strains	1, 2	Docking	LasR, PqsR, RhlR	Psoralen
[50]	PAO1	1, 2	Docking	LasI, LasR	*Cassia fistula* extract
[51]	PAO1	1, 2	Docking	LasR, QscR, LasI	Synthetic xanthine derivative
[52]	None	*2*	VS, Docking, MD	LasR	Molecule database
[53]	PAO1	1, 2	Docking	PqsR	Vitamins E and K1
[54]	PA14	1, 2	VS, docking	LasI, LasR	Phytochemical database
[55]	PA AM26	1, 2	Docking, MD	LasR, PhzR, RhlR	Literature
[56]	PA P8, P6	1, 2	Docking	LasI, LasR, RhlI	Literature (phytochemicals)
[57]	PAO1	1, 2	Docking	LasR, RhlR, PqsR, LasB	Literature (synthetic compounds)
[58]	PAO1	1, 2	Docking	LasR, PqsR, RhlI	Synthesis of activated furan compounds
[59]	PAO1	1, 2	Docking	LasR	Phytochemical database
[60]	PA MTCC2488	1, 2	Docking	LasI LasA	Crude biosurfactants from *Lactiplantibacillus plantarum* (bacteria)
[61]	PAO1	1, 2	Docking	LasR	Phenyloxadiazole sulfoxide derivatives
[62]	Clinical isolate of PA	1, 2	Docking	LasR, QsIA	Emodin and gallic acid
[63]	PA ATCC 27853	1, 2	Docking, MD	QscR, LasR, pqsR, RhiR, MexB, Ampc	6-gingerol
[64]	PAO1	1, 2	Docking	LasR, RhIR	Literature
[65]	None	*2*	VS, MD	LasR	Natural food compound database
[66]	PAO1-L WT, PAO1 pqsA CTXluxHpqsA	1, 2	Docking, MD	PqsR	FDA-approved database
[67]	PA ATCC 15442	1, 2	Docking	LasI, PqsA	Literature
[68]	PAO1	1, 2	Docking, MD	LasI, LasR, LasA, PqsR RhIR, PiiT, PilY1	Coumarin
[69]	PAO1	1, 2	Docking	QscR	Prazosin
[70]	PA	1, 2	Docking	ExoS, ExoT, ExoU	Literature
[15]	N/D	*2*	Docking, MD	PqsA	Literature
[71]	N/D	*2*	Docking, MD	LasR, PqsR, RhIR	Marine metabolites database
[72]	N/D	*2*	Docking, MD, ADMET	Las R, PqsR, RhIR	Marine metabolites with a furanone backbone
[73]	N/D	*2*	VS, docking, AMDET	LasI, LasR, LasA,	Bioactive natural compounds database
[74]	N/D	*2*	VS, docking, MD, AMDET	LasR	Synthetic and natural compound databases
[75]	N/D	*2*	Docking	LasI, LasR, Rhll, RhlR	Nanoparticles
[76]	PA	1, 2	Docking	LasR	Flavone derivative
[77]	Clinical isolates of PA	1, 2	Docking	LasI, PqsR	Methyl gallate from *Mangifera indica*
[78]	N/D	*2*	Docking, MD	LasI, LasR, PqsE, Vfr, QScR, MvfR	AHL-analogs derived from plant metabolites

PA: *Pseudomonas aeruginosa*; N/D: not data (exclusive term for in silico studies), 1: in vitro; 2: in silico; MD: molecular dynamics; VS: virtual screening; ADMET: absorption, distribution, metabolism, excretion, and toxicity.

**Table 2:** Structural information of quorum-sensing-related proteins from *Pseudomonas aeruginosa* used in silico analyses in the selected studies.

Protein	PDBID	Resolution (Å)	General Stoichiometry	Sequence Length (aa)	Reference
LasI	1RO5	2.3	Monomer	201	[15,35,36,41,44,48,50,51,54,56,60,67,68,73,75,77,78]
LasR	2UV0	1.8	Homo 2-mer	175	[39,41,43,44,45,46,48,50,54,55,56,57,58,59,64,68,69,75,76,78]
	6V7X	2.9	Hetero 3-mer—A2B1	A: 69 B: 239	[36,37,40,42,71,72]
	6MVN	2.2	Homo 2-mer	239	[51]
	6D6A	1.9	Homo 2-mer	170	[64]
	6D6L	1.63	Homo 2-mer	170	[64]
	6D6O	1.65	Homo 2-mer	170	[64]
	6D6P	1.65	Homo 2-mer	170	[64]
	4NG2	2.41	Hetero 3-mer- A2B1	A: 184 B: 113	[35,62]
	3IX3	1.4	Homo 2-mer	173	[38,49,74,76]
	3IX4	1.8	Homo 2-mer	173	[52,65]
RhlR	8DQ0	3.74	Hetero 4-mer—A2B2	A: 241 B: 301	[37,45,57]
	4Y15	2.8	Homo 2-mer	246	[46,64,72]
	4Y17	2.84	Homo 2-mer	246	[64]
	P54292	-	N/D		[68]
	7R3H	3.49	Homo 2-mer	241	[65]
	3D modeling	N/D	N/D	N/D	AlphaFold: [35,36,49,55], SWISS-MODEL ([41,56,58,71])
PqsR	4JVD	2.95	Homo 2-mer	216	[36,46,47,53,57,65,71,77]
	4JVI	2.9	Homo 2-mer	216	[58,66,68]
	6B8A	2.65	Homo 2-mer	203	[39,49]
	5OE3	1.43	Monomer	407	[67]
	8DQ0	3.74	Hetero 4-mer- A2B2	A: 241 B: 301	[45]
RhlI	3D modeling	N/D	N/D	N/D	AlphaFold [35], SWISS-MODEL [41,56]
QscR	3SZT	2.55	Homo 2-mer	237	[41,65,69]
	6CC0	2.5	Homo 2-mer	237	[51,78]
PqsA	5OE3	1.43	Monomer	407	[15,39,41,43,67]
PqsE	7TZA	2.1	Monomer	304	[39]
PPqsE	4JVC	2.5	Homo 2-mer	216	[75,78]
LasA	3IT7	2.14	Monomer	182	[48,60,68]
LasB	3DBK	1.4	Monomer	301	[48,57]
LpxC	2VES	1.9	Homo 3-mer	299	[38]
EsaI	1KZF	1.80	Monomer	230	[68]
PilT	3JVV	2.60	Homo 6-mer	356	[68]
PilY1	3HX6	2.10	Homo 2-mer	570	[68]
ExoS	1HE9	2.40	Homo 2-mer	134	[70]
ExoT	6GNN	3.70	Hetero 4-mer—A2B2	A: 248 B: 246	[70]
ExoU	4AKX	2.95	Hetero 4-mer—A2B2	A: 127 B: 660	[70]
Pseudaminidase	2W38	1.90	Homo 3-mer	438	[78]
Vfr	2OZ6	2.8	Homo 2-mer	207	[78]

N/D: not data.

**Table 3:** Top-ranked ligands targeting quorum-sensing-related proteins in *Pseudomonas aeruginosa* identified from selected studies based on docking binding affinity.

Rank	Ligands	Origin of Ligands	Target	Affinity Energy (Kcal/mol)	Amino Acid Interaction Residues	References
1	a	Zinc15 database	LasR	−14.5	H-bond: Tyr56, Ser129 π-interactions: Tyr56, Tyr64, Trp88, Phe101	[65]
2	Lupiwighteone	Zinc15 database	LasR	−13.3	H-bond: Trp 60, Leu 110 π-interactions: Tyr 64, Trp 88, Phe 101	[65]
3	Shinpterocarpin	Zinc15 database	LasR	−13.3	H-bond: Leu110 π-interactions: Tyr64, Trp88, Phe101	[65]
4	(R)-Glabridin	Zinc15 database	LasR	−13.2	π-interactions: Tyr64, Trp88, Phe101	[65]
5	c	Synthetic and natural compound databases	LasR	−11.35	H-bond: Tyr56, Ser129, Asp73, Trp88	[74]
6	Chrysin	Isolated compounds purchased	LasR	−11.0	H-bond: Asp73, Arg61 π-interactions: Ala36, Leu40, Tyr47, Ala50, Tyr64, Val76, Leu125, Ala127	[56]
7	d	Synthetic and natural compound databases	LasR	−10.71	H-bond: Tyr56, Ser129, Asp73, Trp88	[74]
8	Luteolin	Isolated compounds purchased	LasR	−10.6	H-bond: Gly 38, Aeg61, Asp73, Ser129 π-interactions: Leu36, Tyr47, Tyr56, Trp60, Val76, Cys79, Phe101, Leu125, Ala127 C-H bond: Try64	[56]
9	e	Synthetic and natural compound databases	LasR	−10.31	H-bond: Tyr56, Ser129, Asp73, Trp88	[74]
10	Rhein	*Cassia fistula* extract	LasR	−10.26	H-bond: Arg61, Thr75, Thr115, Ser129 π-interactions: Leu36, Ile52, Tyr56, Tyr64, Val76, Trp88, Ala127	[50]
11	6-gingerol	Extract from the seeds of *Aframomum melegueta*	QscR	−10.15	H-bond: Ser38, Gly40, Tyr58 π-interactions: Tyr66, Phe101 Hydrophobic interactions: Leu80, Lue102, Val131, Val143, Val148, Ala165	[63]
12	Benzyl benzoate	Literature	LasR	−10.1	H-bond: Tr 115 Hydrophobic interactions: Leu 110, Phe 101, Ala 105, Trp 88, Leu 36, Tyr 64, Asp 73,	[71]
13	6-gingerol	Extract from the seeds of *Aframomum melegueta*	LasR	−9.71	H-bond: Tyr56, Leu125 π-interactions: Tyr56, Trp60 Hydrophobic interactions: Leu36, Ile52, Val76, Leu110, Ala127	[63]
14	Zinc oxide nanoparticles	Nanoparticles	LasR	−9.58	H-bond: Asn141	[41]
15	Prazosin	Synthetic compound	QscR	−9.5	π-interactions: Ser38, Arg42, Phe54 Hydrophobic interactions: Ser38, Phe54, Tyr58, Tyr66, Val78, Met127	[69]
16	Phylloquinone	Sunflower seeds’ oils	LasR	−9.4	H-bond: Tyr56, Trp60, Ser129 Other Significant Bonds: Leu40, Tyr47, Ala50, Tyr64, Ala70, Asp73, Val76, Cys79, Leu105, Leu110, Leu125, Ala127	[48]
17	Rutin	Isolated compounds purchased	PqsA	−9.4	H-bond: Gly279, Gly300, Thr304, Glu305, Asp382, and His394 Hydrophobic interactions: Pro281, Ala278, Gly302, Ile301, Ala303, Tyr378, Thr164, and Ser280	[67]
18	f	Literature	PqsA	−9.3	Pro205, Phe252, Met213, Gly214, Phe209, Ala278, Gly279, Tyr211, Gly210, Ser280, Val309, His308, Thr304, Ser165, Gly302, Ala303, Thr164, His394, Ser167, Glu305, Lys172, Thr168, Arg397, Asp382, Thr380	[15]
19	Zinc oxide nanoparticles	Nanoparticles	LasI	−9.16	H-bond: Gly116	[41]
20	Miconazol	Synthetic antifungal agent	LasR	−9.06	H-bond: Arg61 Hydrophobic interactions: Leu36, Gly38, Leu39, Leu40, Ala50, Ile52, Thr75, Val76, Cys79, Thr80, Leu125, Gly126, Ala127 π-interactions: Tyr56, Trp60	[57]

a: 3,3′-Dihydroxy-4,5-dimethoxybibenzyl. c: N-[2-[2-(3-Bromophenoxy)ethylsulfanyl]ethyl]pyrrolidine-2-carboxamide. d: 2-Amino-N-[2-[2-(3-bromophenoxy)ethylsulfanyl]ethyl]pentanamide. e: (2S)-2-Amino-N-[2-[2-(3-bromophenoxy)ethylsulfanyl]ethyl]-3-methylbutanamide. f: 6-amino-9-((2R,3R,4S,5R)-3,4-dihydroxy-5-(((N-(3-hydroxy-2-naphthoyl)sulfamoyl)oxy)methyl)tetrahydrofuran-2-yl)-3H-purine-1,7,9-triium.

**Table 4:** Experimental assays reported in the selected studies targeting quorum-sensing in *Pseudomonas aeruginosa*.

Types of Studies in Addition to In Silico	Number of Publications (%)	Reference
Growth inhibition (MIC, MBC and/or Diffusion)	27 (81.8%)	[35,36,38,39,40,41,42,44,45,46,47,48,49,50,51,53,55,56,57,58,60,62,63,64,66,76,77]
Biofilm Inhibition	25 (75.7%)	[35,36,40,41,42,43,44,46,47,48,49,50,51,53,55,56,57,58,59,60,62,63,64,66,76,78]
Virulence Inhibition (Pyocyanin, Pyoverdine, Protease, Elastase and/or Alginate)	24 (72.7%)	[35,39,40,43,44,45,46,47,48,49,50,51,53,55,56,57,58,59,60,61,64,66,69,76,78]
Motility (Swimming, Swarming and/or Twitching)	15 (45.4%)	[35,41,43,44,46,48,49,50,51,56,60,63,64,66,76]
Gene Expression (RT-qPCR and/or RT-PCR)	9 (27.2%)	[35,45,46,49,50,51,57,58,63,76]
Microscopy (MCF and/or MCB)	9 (27.2%)	[41,43,44,45,46,48,50,59]
Animal Model	4 (12.2%)	[51,57,63]
*Caenorhabditis elegans*	3 (9.1%)	[49,50,58]

**Table 5:** Ligands with higher docking binding affinity targeting quorum-sensing-related proteins in *Pseudomonas aeruginosa* and their associated experimental effects, according to the selected studies.

Rank	Ligands	Target	Affinity Energy (Kcal/mol)	MIC (mg/mL)	% Swimming Inhibition	% Swarming Motility	% Biofilm Formation Inhibition	% Total Protease Inhibition	% Inhibition of Elastase (LasB)	% Pyocyanin Inhibition	% Inhibition of Rhamnolipids	References
1	Chrysin	LasR	−11.0	0.009	45 (1/2 MIC)	40 (1/2 MIC)	41 (1/2 MIC)	N/D	N/D	N/D	N/D	[48]
2	Luteolin	LasR	−10.6	0.08	50 (1/2 MIC)	40 (1/2 MIC)	36 (1/2 MIC)	N/D	N/D	N/D	N/D	[48]
3	Rhein	LasR	−10.26	N/D	N/D	76.7 (0.15 mg/mL)	51.4 (0.15 mg/mL)	64 (0.15 mg/mL)	75 (0.15 mg/mL)	72 (0.15 mg/mL)	43 (0.15 mg/mL)	[50]
4	Zinc oxide nanoparticles	LasR/LasI	−9.58/−9.16	1.6	<45 (300 mg/mL)	62.80 (300 mg/mL)	57.08 (300 mg/mL)	N/D	N/D	N/D	N/D	[41]
5	Rutin	PqsA	−9.4	0.5	N/D	N/D	73.7 (0.25 mg/mL)	N/D	N/D	N/D	N/D	[67]

N/D: not data.

## Data Availability

The original contributions presented in this study are included in the article. Further inquiries can be directed to the corresponding author.

## Abbreviations

The following abbreviations are used in this manuscript:

PA	*Pseudomonas aeruginosa*
QS	Quorum-Sensing
AHL	Acyl-Homoserine Lactone
VS	Virtual Screening
MD	Molecular Dynamics Simulations
ADMET	Absorption, Distribution, Metabolism, Excretion, and Toxicity
IQS	Integrated Quorum-Sensing
MIC	Minimum Inhibitory Concentration
MBC	Minimum Bactericidal Concentration
PRISMA	Preferred Reporting Items for Systematic Reviews and Meta-Analyses
GC-MS	Gas Chromatography–Mass Spectrometry
HPLC	High-Performance Liquid Chromatography
TLC	Thin-Layer Chromatography
